# The Effects of Severe and Frequent Back Pain on Mental Health: Does Perceived Socioeconomic Status Matter?

**DOI:** 10.1093/geroni/igaa057.1285

**Published:** 2020-12-16

**Authors:** Christina Mu, Dylan Jester, Peggy Cawthon, Katie Stone, Soomi Lee

**Affiliations:** 1 University of South Florida, Tampa, Florida, United States; 2 California Pacific Medical Center, San Francisco, California, United States

## Abstract

Back pain and mental health are related. The relationship may differ by socioeconomic status (SES); yet, research has not examined the moderating role of perceived SES. We examined if the association between back pain and poor mental health is more pronounced for older men with lower perceived SES. We used a sample of community-dwelling older men (>65yrs) with back pain from the Osteoporotic Fractures in Men Study (n=4,035). Participants reported their perceived SES in comparison to others in the community and in the nation (1=lowest—10=highest), back pain severity (mild—severe), and frequency (rarely—all of the time). Mental health was assessed with the 12-item Short Form Health Survey. Analyses were adjusted for sociodemographic and health covariates. Greater pain severity and higher pain frequency were associated with poorer mental health (p<.001). Only severe pain was associated with poorer mental health (p<.001). Pain ‘some of the time’ (p=.02), ‘most of the time’ (p=.02), and ‘all of the time’ (p=.001) were associated with poorer mental health. Adverse effects of pain were reduced with greater community SES (p<.001 for severe pain; p=.02 for ‘all of the time’ pain frequency) and greater national SES (p=.01 for severe pain; frequency n.s.). Reports of pain were worse for individuals with lower SES. Adverse associations of severe and high frequency back pain with poor mental health are more apparent in older men with lower perceived SES. Where one ranks oneself within their community or nation can influence the pain and mental health link.

